# St. George's Hospital

**Published:** 1829-08

**Authors:** 


### ST. GEORGE'S HOSPITAL.

### *Extensive Disease of the Arterial System. Rupture of the Right common Iliac Artery. Peritonitis, &c.*

On the 27th of May last, the body of Elizabeth Hancock, sixty years of age, was examined.

The right leg was swollen and pitted on pressure, and its two lower thirds presented patches of livid discoloration of the skin, which also extended in a slight degree along the front of the thigh to the groin. The appearance was not that of mortification; for the whole circumference of the limb was not affected, and the deeper textures were perfectly sound, with the exception of the subcutaneous cellular membrane, which was infiltrated with serum and a little pus.

Abdomen: The peritoneum investing the liver, stomach, and indeed many other portions of the viscera, had shreds of coagulable lymph attached to it. There was no pus, little or no serous effusion into the abdomen, and the general peritonitis was but slight.

In the right iliac and inguinal regions, the pelvic cavity, and more especially on the right side of the latter, the inflammation of the peritoneum was more intense, and a couple of ounces of brown turbid fluid, apparently a mixture of serum and pus, had collected in the basin of the pelvis. In this situation the parts were all dark and modena-coloured, and, on dissecting the peritoneum from the abdominal muscles in the right groin, portions of coagulum, varying in firmness, and mixed with the products of inflammation, were discovered. This appearance on the outside of the peritoneum continued in the cup of the ilium, and particularly around the iliac vessels, which, in fact, were enclosed by a stratum of denser coagulum, equalling their own diameter. It was thought that there might be phlebitis, and the iliac vein was cut out to be examined, but the tube was free from any inflammation.

Attention was now attracted to a dark-coloured tumor on the sacrum, in the situation of the great vessels. The viscera were cleared away, and the aorta and vena cava exposed from the diaphragm to the termination of their iliac divisions. The vena cava was then slit up, and its cavity found to be perfectly free. The tumor noticed before was now clearly proved to be a continuation of that coagulum which had accompanied the iliac artery to the groin, and diffused itself in the iliac and inguinal regions. It was firm, not very recent, and closely invested the vena cava at its lower part; after which it passed over the right common iliac vein, in company with the right common iliac artery and its prolonged external trunk.

The vessels, with the coagulum around them, were removed from the body, and carefully examined on the table. The aorta was universally diseased. In some places, patches of atheromatous matter, in others of cartilaginous, and in others (still more numerous) of actual ossific depositions, were found between the inner and the outer coats. The middle appeared to be the seat of the degeneration. Throughout the whole length of the tube, the internal coat was cracked and fissured in every direction, and the sharp osseous spiculæ projected into its cavity. In a number of places the external tunic alone remained to confine the blood. In the right common iliac, about half an inch from its origin, a nearly complete circle of osseous deposit reduced the caliber of the vessel to less than that of the superficial femoral in the middle of the thigh, and gave it exactly the appearance of a stricture. Immediately beyond this, the artery was dilated into a pouch the size of a filbert, and it seemed to be somewhere hereabouts (though we could not precisely ascertain the spot) that one of the fissures in the vessel had given way, and the blood got abroad into the loose cellular texture external to the peritoneum.

All the branches of the abdominal aorta were diseased in the same way as their parent trunk. The kidneys were wasted; the liver was dark coloured; the stomach healthy; the intestines externally healthy also, but internally not examined.

Thorax: The lungs and pleuræ were free from disease; the pericardium was natural, but its cardiac layer exhibited those white patches so frequently observed. The auricles of the heart were natural, and so were the parietes of the right ventricle, but its cavity was very small. The parietes of the left ventricle were upwards of an inch in thickness, its cavity remaining unaltered; in other words, it was hypertrophied without dilatation. The tricuspid and mitral valves were sound; the semilunar of the aorta were slightly indurated at their attached borders. The coronary arteries were extensively ossified, being reduced in more than one situation to a firm, bony, and contracted ring. The pulmonary artery was perfectly healthy.

The arch of the aorta presented externally a beautifully injected and arborescent appearance of its vasa vasorum. It was greatly dilated at its origin, and its branches, the innominata, left carotid, and subclavian, were also much enlarged in their caliber. The dilated part of the arch, which was also the injected one, was free from ossific or atheromatous depositions, and its internal coat was sound. The remainder of the arch, the branches, and the descending thoracic portion, were equally diseased, and in the same manner as the abdominal.

The head was not examined.

We have given the dissection first, because with that commenced our acquaintance with the case, the previous part of which is involved in much obscurity. The patient applied to Mr. CATES only four days before her death, and we have been informed that the symptoms at that time were vomiting, pulse small and weak, tongue dry and brown, expression of countenance anxious and typhoid.

Mr. Cates sent her into the hospital on the 25th, the second or third day of her illness, where she was visited and prescribed for by the house apothecary, Mr. HUTCHINS, in the absence of Dr. CHAMBERS. She had now great tenderness over the abdomen; hot skin; pulse feeble, and 120; tongue dry and brown; teeth covered with dark sordes; bowels confined. The right leg and foot were the seat of dark erysipelatous inflammation, and purple vesications covered the inner ankle.

Mr. Hutchins ordered Hydrarg. Submur., Pulv. Ant. āā gr. iij. hâc nocte. Ol. Ricini ʒiv.

26th.—Infus. Ros. ℥iss.; Quin. Sulph. gr. iss.; Tinct. Op. ♏v. quartâ quâque horâ.—Spir. Tenuior. ℥ij.; Liq. Ammon. acet. ℥iv.; Mist. Camph. ℥vi. M. fiat lotio cruri assidue appl.

In the evening a blister was placed on the abdomen, and an effervescing saline ordered to be taken every four hours.

On the 27th she died.

This dissection is interesting in several points of view. It shows how extensively diseased the arterial system may be, consistently with a tolerable performance of the vital and corporeal functions; and it illustrates a fact which we have often noticed, namely, the existence of hypertrophy of the left ventricle of the heart, along with disease of the coats of the blood-vessels. The smallness of the rupture in the common iliac will account for its not having killed *instanter*, by the sudden and profuse effusion of blood; and the greater consolidation of the coagulum immediately around the vessels, than of that in the groin and other situations, proves that the extravasation was slow and gradual.—*Medical Gazette.*

